# Vertical stratification of P pools in subtropical plantation soils under fertilization and dry–season irrigation: multiomics regulatory strategies

**DOI:** 10.3389/fmicb.2025.1714023

**Published:** 2026-01-05

**Authors:** Shitao Zhang, Yang Mo, Jincheng Yang, Xiaoshan Chen, Meiling Gao, Yan Su, Quan Qiu, Qian He

**Affiliations:** Guangdong Key Laboratory for Innovative Development and Utilization of Forest Plant Germplasm, College of Forestry and Landscape Architecture, South China Agricultural University, Guangzhou, China

**Keywords:** ferralsols, phosphorus cycling, vertical differentiation, multiomics, plantation

## Abstract

The rapid expansion of fast-growing plantations in subtropical regions is closely linked to silvicultural practices, however, improper practices often lead to soil acidification and reduced nutrient bioavailability. Phosphorus (P), one of the most critical elements for plantation tree growth, shows complex spatial distribution patterns in soil that are influenced by multiple factors, directly affecting plantation productivity. This study investigated the effects of long-term fertilization and dry-season irrigation on the vertical distribution of phosphorus in an 8-year-old subtropical *Eucalyptus* plantation. This study employed stratified sampling (0–30 cm topsoil, 30–60 cm subsoil, 60–90 cm substratum) during dry seasons, coupled with metagenomics, metabolomics, and environmental factor analysis, to reveal vertical phosphorus cycling patterns and multiomics regulatory networks. Key findings: (1) Fertilization and dry-season irrigation had a limited influence on labile phosphorus and the diversity of P-cycling microorganisms. The topsoil presented significantly greater P availability than did the subsoil, manifested as elevated acid phosphatase activity (ACP), significant enrichment of the tryptophan metabolic pathway, and greater microbial diversity. (2) pH and the C:P ratio represent critical factors of vertical stratification in soil P cycling. Under acidic conditions, topsoil microorganisms facilitate P release via diverse metabolic pathways, whereas oligotrophic constraints in the substratum limit enzymatic activities. (3) Potential cross-stratum microbial functional coordination exists in acidic soil P cycling, with linkages to tryptophan metabolism and polyphosphate, synthesis/degradation. Our study provides theoretical multiomics insights for optimizing the management of soil P pools in subtropical plantations under fertilization and dry-season irrigation.

## 1 Introduction

The establishment of large-scale plantations underpins the sustainability of modern forestry. However, the growth of fast-growing plantations typically depletes substantial soil nutrients and water. During the subtropical dry season, irrigation becomes critically important. Inappropriate fertilization and dry-season irrigation not only increase costs and reduce economic returns but also decrease soil nutrient availability, exacerbate acidification and compaction (Zheng et al., 2015), and induce long-term soil degradation (Liang et al., 2022), as observed in regions such as the Amazon Basin, Southeast Asia, Central Africa, eastern Australia, and southern China. Understanding the distribution patterns and underlying mechanisms of nutrient cycling in ferralsols is essential for enhancing soil fertility, guiding efficient silviculture, and maintaining ecosystem health.

Phosphorus (P), a nonrenewable nonmetallic mineral resource, critically influences productivity and ecosystem stability in plantation soils. The soils in Eucalyptus plantations are predominantly acidic. In acidic soils, P is predominantly immobilized by iron and aluminum oxides, reducing its bioavailability and restricting root uptake (Cernusak et al., 2010). Acid-tolerant microbes paradoxically thrive at low pH, activating P cycling via specialized metabolic pathways to meet bioavailable P demand (Siles et al., 2022). Notably, excessive P fertilization exacerbates fixation by suppressing both organic P mineralization and inorganic P solubilization (Liu C. et al., 2024). Paradoxically, certain acid-tolerant microorganisms thrive under low-pH conditions, employing specialized metabolic pathways to increase P mobilization, whereas excessive P inputs exacerbate P fixation by suppressing organic P mineralization and inorganic P solubilization. Despite vertical stratification in terms of P distribution and speciation, research has focused predominantly on topsoil due to sampling challenges, high costs, and limited deep-layer P content. At least three mechanisms underpin the need to consider substratum P migration (Achat et al., 2013; Ma et al., 2024). (1) In sandy soils with shallow-rooted trees, reduced Fe/Al oxide adsorption facilitates P leaching (Fujii et al., 2021). (2) Microbial degradation of recalcitrant substratum P via rhizodeposits contributes to long-term P reserves (Achat et al., 2012). (3) Preplanting amendments (e.g., slow-release fertilizers) increase deep-layer P availability by releasing Fe/Al-bound P. Addressing these knowledge gaps is vital for constructing a holistic framework of vertical P cycling to improve plantation productivity (Ch’ng et al., 2014).

Microbial communities and rootledge gaps is vital for constructing a holistic framework of vertical P cyclingtability. Nitrogen fertilization directly amplifies microbial P demand, potentially intensifying P limitation (Zeng et al., 2024). The functional genes governing microbial P cycling include those involved in organic P mineralization (*phnM*, *phoA*), inorganic P solubilization (*gcd*), regulation (*phoU*, *phoR*), transport (*ugpA*, *pstS*), polyphosphate synthesis (*ppk1*, *ppaC*), and degradation (*HDDC3*, *ppgK*) (Bergkemper et al., 2016). Soil management practices shape phosphatase gene redundancy (intergroup redundancy≈1), highlighting their central role in P cycling (Siles et al., 2022). Gene expression shifts reflect metabolic reprogramming in nutrient cycling. Nitrogen fertilization upregulates *amoA*, *nirK*, and *phoD*, activating metabolic pathways to enrich uracil, guanine, and indole while suppressing D-phenylalanine, a strategy that enhances rhizosphere functionality (Gu et al., 2023). Nevertheless, significant knowledge gaps persist regarding microbial communities linked to soil P cycling, functional gene dynamics, and metabolic variations in plantation systems. In particular, long-term silviculture reshaped the substratum microbiota and metabolism, potentially redefining P cycling pathways (Liu S. et al., 2024).

This study aims to elucidate the vertical stratification patterns of P cycling in *Eucalyptus* plantations, providing a foundation for sustainable plantation management. We hypothesize associations between multiomics factors and soil phosphorus stratification. The objectives of this study are as follows: (1) Characterize the vertical stratification patterns of P fractions under fertilization and dry-season irrigation; (2) identify key microbial taxa and metabolic pathways that mediate vertical stratification in acidic soil P cycling; and (3) elucidate vertical stratification patterns via P cycling functional networks spanning microbe–gene–metabolite axes. To test this hypothesis, we conducted experiments in ferralsols of *Eucalyptus urograndis* plantations in southern China (Zhu et al., 2019). *Eucalyptus urograndis*, a hybrid of *Eucalyptus urophylla* and *Eucalyptus grandis*, is widely cultivated for its high productivity and adaptability (Wang et al., 2022; de Barbosa et al., 2023). Soil properties across layers and treatments were analyzed via Illumina sequencing, LC–MS/MS, coexpression networks, and DIABLO-based multiomics integration.

## 2 Materials and methods

### 2.1 Site description and experimental design

The experiment was conducted at the South China Agricultural University Teaching and Research Base in Zengcheng District, Guangzhou (23°14′57″N, 113°38′31″E). The experimental forest was terraced to reduce soil erosion, covering a total area of 11,700 m^2^ (Yu et al., 2019). This region has a subtropical monsoon climate, with seasonal drought occurring from October to March. An analysis of variance (ANOVA) conducted on the mean precipitation during the dry season, wet season, and annual total from 2017 to 2024 revealed that the dry-season precipitation was significantly lower than that of both the wet season and the annual average, thereby providing empirical support for dry-season water supplementation (Table 1). Soil sampling was conducted in December 2024 (dry season), during which the mean monthly precipitation was 0 mm. The soil is classified as ferralsol, with an average pH of 4.88 and a total P content ranging from 160 to 240 mg/kg. High acidity and limited P availability constrained plantation productivity. In April 2017, 3-month-old *Eucalyptus urograndis* seedlings (uniform height) were planted at a density of 2 × 3 m. Four treatments were implemented: fertilization with dry season irrigation (FDS), dry season irrigation alone (DS), fertilization alone (F), and a control with no silvicultural measures (CK). Each treatment included five east–west–oriented plots per terraced unit, with four north–south-oriented treatment plots per unit, totaling 20 plots across five terraced units. Nine seamless PVC panels (6 mm thick, 1.5 m deep) of adequate length were installed to hydraulically isolate the plots, thereby preventing subsurface cross-contamination of fertilizer-derived nutrients and irrigation water throughout the trial. For the FDS and F plots, 400 g of *Eucalyptus*-specific fertilizer (Guangdong Dayi Agricultural and Forestry Ecological Technology Co., Ltd.) was applied annually for 4 years at four positions (40 cm east, west, south, and north of each tree), delivering 45 g N, 21 g P, 24 g K, 0.3 mg B, and 0.15 mg Zn per tree per application. In the FDS and DS plots, drip irrigation pipes were buried 40 cm from each tree at a depth of 40 cm, providing 32 L of water weekly during the dry season (8 h per week). To ensure consistent annual irrigation volume and fertilization rate (during the first 4 years) across respective treatments. On December 2, 2024, all the trees were measured for height and diameter at breast height (DBH). The mean height and DBH were calculated as standard parameters, with one representative tree per plot marked for further analysis.

**TABLE 1:** Results of ANOVA and tukey tests for mean annual rainfall across dry season, wet season, and full year (2017–2024).

Time period	df	Mean	SE	Tukey grouping
Dry-season	2	2.44641	0.55425	C
Wet-season	2	11.27132	1.25308	A
Annual	2	7.3144	0.93419	B

Different letters indicate significant differences among treatments at *p* < 0.05 (multiple comparisons with Tukey tests). Meteorological data were obtained from Meteostat (https://meteostat.net/en/place/cn/guangzhou?s=59287&t=2017-01-01/2025-03-31; accessed on 2 October 2025).

### 2.2 Soil sampling

On December 21, 2024, soil profiles (1 m depth) were excavated within 40 cm of tree trunks from each standard tree. For each of the four treatments within the terraced units, sampling positions (east, west, south, north) were randomly selected, ensuring alignment with the fertilization and drip irrigation sites. A generalized stratification based on soil-forming processes was employed (Xu et al., 2024), dividing profiles into three genetic horizons: topsoil (0–30 cm, dominant processes: organic matter accumulation and eluviation), subsoil (30–60 cm, characterized by illuviation), and substratum (60–90 cm, primary influence: parent material). This stratification was used to test the hypothesis that the soil phosphorus pool exhibits stratified differences. Soil samples (1 kg per layer) were collected using the quartering method, yielding a total of 60 samples from 20 plots. Each treatment included five replicates per soil layer. The samples were divided into three subsets: (1) stored in aluminum boxes for moisture determination; (2) air-dried, sieved through 0.25 mm sterile mesh, and bagged for chemical analysis, P fractionation, and enzyme activity assays related to soil P cycling; and (3) transferred into two batches of 50 mL sterile centrifuge tubes and preserved at -80 °C for subsequent metagenomic and metabolomic analyses.

### 2.3 Analysis of soil abiotic environmental factors

The soil water content (WC) was determined gravimetrically via oven drying. Total nitrogen (TN) was quantified via the Kjeldahl digestion method. The soil organic carbon (OC) content was calculated by dividing the soil organic matter content, measured via dichromate-graphite digestion, by a factor of 1.724. The soil pH was determined potentiometrically. Five enzyme classes mediate P cycling by phosphate-solubilizing microorganisms, including acid phosphatase (ACP), alkaline phosphatase (ALP), phytase (PHY), ribonuclease (RNase), and pyrophosphatase (PPase). ACP and ALP activities were determined via disodium phenyl phosphate colorimetry with distinct buffers. PHY and RNase activities were quantified by measuring inorganic P release from sodium phytate and ribonucleotide substrates, respectively. Soil pyrophosphatase (PPase) activity was determined by quantifying the enzymatic hydrolysis of pyrophosphate to phosphate, with the absorbance measured spectrophotometrically at 700 nm.

A modified Hedley sequential extraction method was employed to quantify soil available P (Hedley and Stewart, 1982). Acidic soil was extracted with NH_4_F-HCl solution, and the extracted P was quantified via the molybdenum-antimony colorimetric method. For P fractionation, samples were digested with H_2_SO_4_-H_2_O_2_ and sequentially extracted with 0.5 M HCl, 0.5 M NaHCO_3_, 0.1 M NaOH, 1 M HCl, H_2_O_2_, concentrated H_2_SO_4_, and anion-exchange resin. Orthophosphate concentrations were determined colorimetrically under acidic conditions (700 nm), yielding nine P fractions: Resin-Pi, NaHCO_3_-Pi, NaHCO_3_-Po, NaOH-Pi, NaOH-Po, D-HCl-P, and C. HCl-Pi, C. HCl-Po, and residual-Pt. These fractions were categorized, integrated, and recalculated into P cycling indices on the basis of prior studies to elucidate their ecological significance (Table 2 and Supplementary Table 1).

**TABLE 2:** Abbreviations table.

Index of phosphorus cycle	Abb.	Index of phosphorus cycle	Abb.
Resin-Pi	–	Nitrogen to phosphorus ratio	N:P
D-HCl-P	Ca-P	Soil organic carbon to nitrogen to phosphorus ratio	C:N:P
Available phosphorus	AP	Organic phosphorus mineralization potential	OPP
Iron and aluminum-bound phosphorus	Fe/Al-P	Total phosphorus	TP
Labile phosphorus pool	LPP	Carbon to phosphorus ratio	C:P
Phosphorus activation efficiency	PAE	Potentially available phosphorus	PAP

### 2.4 Analysis of soil abiotic environmental factors

Dry season samples were subjected to multiomics analysis. We used the FastPure Soil DNA Isolation Kit (Omega Biotek, Norcross, GA, United States) to extract nucleic acids. The extracted nucleic acids were quantified via TBS-380 and NanoDrop2000. The quality of the nucleic acids was assessed via agarose gel electrophoresis via an electrophoresis apparatus (JY600C). The extracted DNA was fragmented to an average size of approximately 350 base pairs (bp) via a Covaris M220 (Gene Company Limited, China). The paired-end library was constructed via NEXTFLEX Rapid DNA-Seq (Bioo Scientific, Austin, TX, United States). Metagenomic sequencing was subsequently performed via the Illumina NovaSeq sequencing platform provided by Shanghai Majorbio Bio-Pharm Technology Co. The sequence insert size was between 420 and 460 for all the samples. The raw sequences were quality controlled via fastp software (including the removal of reads whose length was < 50 bp or whose PHRED score was < 20). Macrogenome assembly of the sequences postquality control (QC) was conducted via MEGAHIT software. A process of refinement was then initiated, which involved the elimination of contig sequences shorter than 300 bp. The coding regions in the assembled genome were identified via Prodigal software with default parameters, followed by genes with lengths > 100 bp, which were subsequently translated into protein sequences. The construction of non-redundant genomes was performed via CD-HIT software (Version 4.6.1),^1^ which has 90% sequence identity and 90% coverage. High-quality reads were subsequently aligned to the non-redundant genomes to calculate gene abundance with 95% identity, employing the SOAPaligner (Version 2.21).^2^ The translated protein sequences were subjected to comparative analysis with the Non-redundant Protein Sequence Database (NCBI-NR) database via Diamond (Version 2.0.13) software to classify the taxonomic reads and calculate species abundance. The KEGG Orthology (KO) assignments and subsequent KEGG metabolic pathway predictions were inferred based on the results of the DIAMOND alignments. The analysis used an *e*-value threshold of 10^–5^ for these alignments. We used the KEGG Mapper—Search&Color Pathway tool^3^ to analyze the distribution of the identified genes/metabolites.

Metabolites were extracted from the soil samples via a methanol–water solvent system (80:20, v/v). Briefly, the samples were processed by grinding, ultrasonication, and centrifugal filtration at low temperature. The filtrate was dried under nitrogen gas and reconstituted in 100 μL of the initial mobile phase (98% H_2_O, 2% acetonitrile, 0.1% formic acid) for LC–MS analysis. Quality control (QC) samples were prepared by pooling equal volumes of all samples to monitor system stability. LC–MS/MS analysis was conducted on the metabolites via a Thermo Fisher Ultra High Performance Liquid Chromatography Tandem Fourier Transform Mass Spectrometry UHPLC-Exploris480 platform. The raw data were processed via Progenesis QI software (Waters Corporation, Milford, United States). Putative metabolite identification was achieved by matching accurate mass (mass error tolerance < 10 ppm) and MS/MS fragmentation spectra against the Human Metabolome Database (HMDB, version 5.0) and METLIN (version 2021). Multivariate statistical analysis, including principal component analysis (PCA) and orthogonal partial least squares-discriminant analysis (OPLS-DA), was performed to discriminate metabolic profiles. Features with variable importance in projection (VIP) scores > 1.0 and *p* < 0.05 (Student’s *t*-test) were considered statistically significant.

### 2.5 Statistical analyses

The data were initially processed and organized via the WPS Office 2024. Statistical analyses were performed in R-4.4.2 with the following packages. Differentially abundant metabolite analysis and VIP value calculation via the ropls package. Differential microbial and gene analyses via the stats package. Redundancy analysis (RDA) and RDA visualization of microbial–environmental relationships via the vegan package. Spearman correlation analysis and heatmap generation via the pheatmap package. LefSe analysis and random forest modeling were used to identify keystones across microbial, metagenomic, and metabolomic datasets via the microeco and randomForest packages. Multiomics integration and modular network analysis via mixOmics and BioManager packages.

The vertical stratification mechanisms of P cycling in acidic soils under fertilization and dry-season irrigation were analyzed via the Stats package in R and the SciPy package in Python. FDR-adjusted *P*-values < 0.05 were considered statistically significant. Origin 2024 generated vertical stratification plots of soil P fractions and enzyme activities. Co-occurrence networks were constructed and visualized via Gephi 0.10.1 and the R package “igraph.” Core structural equation models (SEMs) based on DIABLO multiomics results were developed with the R packages “lavaan” and “semPlot.” All vector graphics were standardized and refined in Adobe Illustrator 2023.

## 3 Results

### 3.1 Vertical stratification of soil properties and P indices under fertilization and dry-season irrigation

Four fertilization and dry-season irrigation treatments (FDS, DS, F, and CK) were analyzed on the plantations. Notably, management practices and their interactions with the soil layers had no significant effect on the active phosphorus fractions across either the dry or the rainy season. Labile P parameters constitute the most critical category in P cycling (Table 3).

**TABLE 3:** ANOVA table for the main effects of treatments and their interactions with soil layers.

Effect	Df	Sum Sq	Mean Sq	*F*-value	*P*-value
Treatment (ResinP)	3	0.32	0.1066	0.848	0.478
Treatment*layer (ResinP)	6	0.5223	0.087	2.410	0.058
Treatment (NaHCO_3_-Pi)	3	0.807	0.2689	1.788	0.169
Treatment*layer (NaHCO_3_-Pi)	6	1.2021	0.2003	1.772	0.148
Treatment (AP)	3	1.617	0.5389	1.717	0.183
Treatment*layer (AP)	6	1.195	0.1992	1.526	0.212
Treatment (PAE)	3	0.01189	0.003964	0.473	0.703
Treatment*layer (PAE)	6	0.0399	0.00665	2.303	0.067
Treatment (shannon index)	3	0.03903	0.01301	1.852	0.164
Treatment*layer (shannon index)	6	0.01742	0.00290	0.413	0.862

“*” denotes the interaction effect between the main factors. Df, Degree of freedom; Sum sq, Error sum of squares; Mean Sq, mean squared error.

**FIGURE 1:**
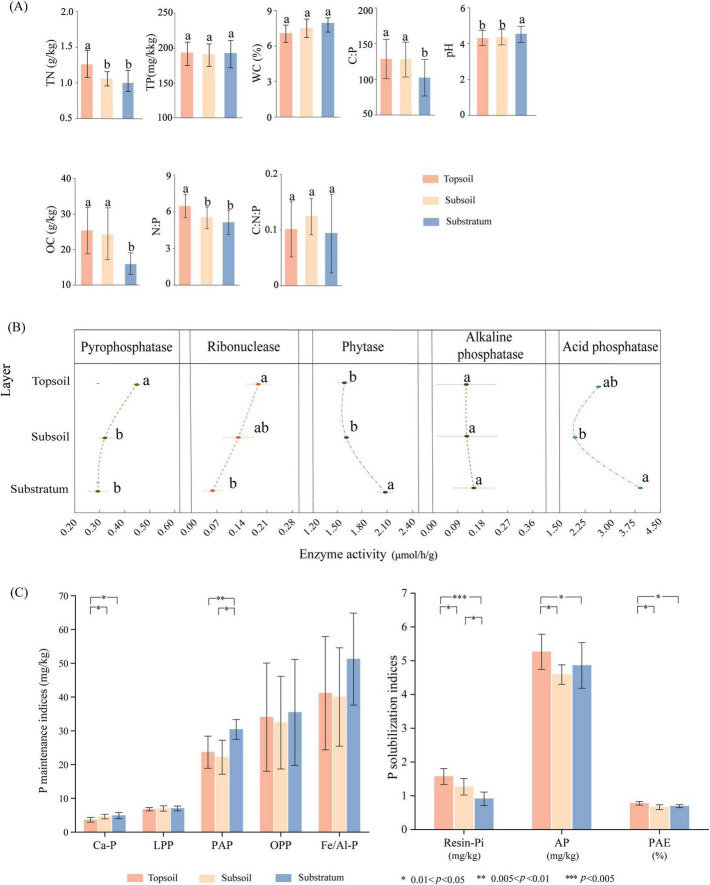
Vertical stratification of soil properties and P indices. **(A)** Soil physicochemical properties, **(B)** P-cycling enzyme activities, **(C)** P indices associated with P cycling. Straight lines adjacent to lowercase letters indicate the standard errors of the mean values (*n* = 12). The asterisk symbol (*) is defined as indicating a significant difference. Asterisks denote statistical significance: *p < 0.05 (significant), **p < 0.01 (highly significant), ***p < 0.005 (extremely significant).

However, significant vertical stratification emerged in the soil properties and P speciation over the 7-year experimental period. During the dry season, the topsoil TN content was significantly greater than that in the subsoil and substratum layers. The substratum presented a higher pH but lower OC content than did the topsoil and subsoil. Compared with those of the topsoil and subsoil, the substratum C:P ratio decreased by 50.49 and 49.30%, respectively, while the topsoil N:P ratio increased by 17.30 and 25.80%, respectively. No significant differences were detected in the TP, WC, or C:N:P ratios across the soil layers (*p* < 0.05) (Figure 1A). Enzyme activities associated with P cycling showed vertical differentiation: PPase, ACP, and RNase activities were higher in the topsoil than in the substratum, with PPase and RNase exhibiting significant differences. In contrast, PHY activity was significantly lower in the topsoil. ALP activity remained minimal and non-significant across layers (*p* < 0.05) (Figure 1B). P fraction analysis revealed distinct stratification: Resin-Pi, AP, and PAE in the subsoil decreased by 19.37, 12.78, and 14.68%, respectively, compared with those in the topsoil, whereas reductions in the substratum reached 42.09, 18.03, and 20.31%, respectively. PAP decreased by 21.20% (topsoil) and 27.06% (subsoil) relative to the substratum, and Ca-P in the topsoil declined by 25.27% (*p* < 0.05) (Figure 1C).

### 3.2 Vertical stratification of P-cycling microbial functions under fertilization and dry-season irrigation

After quality control and assembly, each sample yielded an average of 89,224,401 clean reads, 1,485,817 contigs, and 1,779,924 open reading frames (ORFs). After redundancy removal, the non-redundant gene set comprised 18,037,025 genes with an average sequence length of 441.34 bp. A soil P-cycling gene set (P-cycle) was constructed by filtering the non-redundant genes against prior datasets, resulting in 72,760 genes (average length: 492.99 bp). No significant differences in microbial α diversity related to P cycling were observed across the treatments (Table 3). ANOVA of P-cycling taxa and functional genes revealed significant variations only in *ugpA*, *phoN*, and *Gemmatimonas* (*p* < 0.05) (Table 4). Fertilization may induce *ugpA* expression to alleviate P limitation, irrigation could increase P storage via *Gemmatimonas* enrichment under altered moisture, and their synergy might activate *phoN*. However, the lack of significant differences in labile P fractions (Table 3) and the limited number of significantly different genes/taxa weaken these inferences, necessitating validation through long-term trials.

**TABLE 4:** Multigroup comparisons of P cycling-associated microbial taxa and functional genes exhibiting significant intertreatment differences.

Treatment	*ugpA* relative abundance (%)	*phoN* relative abundance (%)	*Gemmatimonas* relative abundance (%)
CK	0.46b ± 0.04	0.21ab ± 0.04	0.32b ± 0.10
DS	0.62a ± 0.11	0.14b ± 0.04	0.50a ± 0.15
FDS	0.57ab ± 0.18	0.22a ± 0.09	0.33b ± 0.05
F	0.64a ± 0.23	0.17ab ± 0.09	0.38b ± 0.14

Different letters indicate significant differences among treatments at *p* < 0.05 (multiple comparisons with Duncan tests).

**FIGURE 2:**
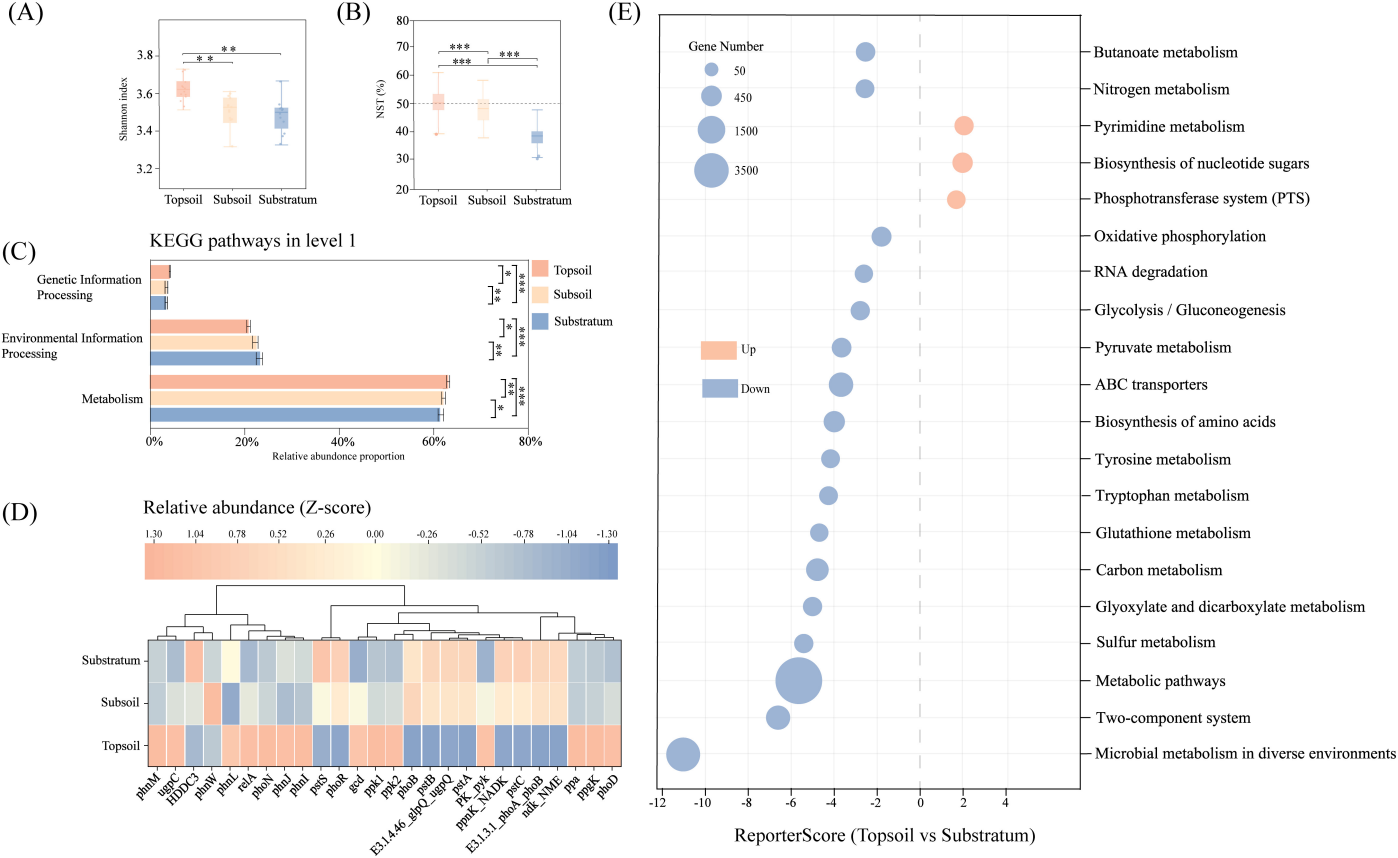
Vertical stratification of microbial assembly and P cycling functions. **(A)** Shannon index comparison of P-cycling taxa across three soil layers. **(B)** NST comparison of P-cycling taxa. **(C)** KEGG Level 1 functional profiling of the P-cycle across layers. Duncan”s test was applied for both **(A–C)** with asterisks denoting statistical significance: *p < 0.05 (significant), **p < 0.01 (highly significant), ***p < 0.005 (extremely significant). **(D)** Comparative analysis of KO terms with significantly differential relative abundances (*p* < 0.05) across three soil layers. The differential relative abundance of identical KOs across distinct soil layers is visualized using color gradients. Prior to this analysis, Z-score normalization was applied to each KO within respective soil strata to amplify inter-layer variations. **(E)** KEGG functional enrichment analysis of differentially abundant KOs between topsoil and substratum. P-cycling-related pathways with significant enrichment (reporter score ≤ 1.65) were identified. Bubble size corresponds to the number of annotated KOs per pathway, while color indicates the upregulation (orange) or downregulation (blue) of pathway-associated KOs.

NR-based taxonomic annotation of the P-cycle set revealed significant vertical stratification in terms of Shannon diversity (*p* < 0.01) (Figure 2A). Normalized stochasticity ratio (NST) analysis was used to quantify the role of vertical stratification in community assembly, revealing a significant decline in NST with soil depth (*p* < 0.0001) (Figure 2B). The NST value of the topsoil approached 50%, indicating that the composition of microbial communities involved in P cycling within acidic topsoil arises from the interplay of short-term environmental disturbances and long-term selection processes, reflecting an equilibrium state. Deterministic processes dominated the subsoil and substratum (NST < 50%). KEGG level 1 functional profiling revealed significant interlayer differences in metabolism, environmental information processing, and genetic information processing (*p* < 0.001) (Figure 2C). These findings demonstrate that the assembly of microbial communities in acidic soils is driven by deterministic mechanisms that directly regulate P cycling functionality.

To elucidate these mechanisms, we first conducted a differential analysis of KEGG Orthology (KO) annotations within the P cycle. The results revealed distinct vertical stratification in microbially mediated soil P cycling pathways. The topsoil presented a relatively high relative abundance of inorganic P solubilization (*ppa*, *gcd*) and polyphosphate synthesis (*ppk1*) genes, whereas substratum upregulated pst transport (pstA, pstB, pstC, pstS) and two-component regulation (phoB, phoR) genes (*p* < 0.001) (Figure 2D). Gene set reporter analysis (GRSA) of topsoil-substratum differential KOs revealed 20 P-cycling-related pathways (reporter score ≥ 1.65) (Figure 2E). Pyrimidine metabolism, the phosphotransferase system (PTS), and the biosynthesis of nucleotide sugars were enriched in the substratum, whereas others dominated the topsoil. These findings suggest that substratum microbes optimize energy efficiency via two-component systems and PTSs under oligotrophic stress, whereas topsoil communities employ diverse metabolic pathways to regulate P equilibrium. Soil P transformation requires further multiangle investigations.

### 3.3 Metabolite-driven vertical stratification of P cycling pathways

**FIGURE 3:**
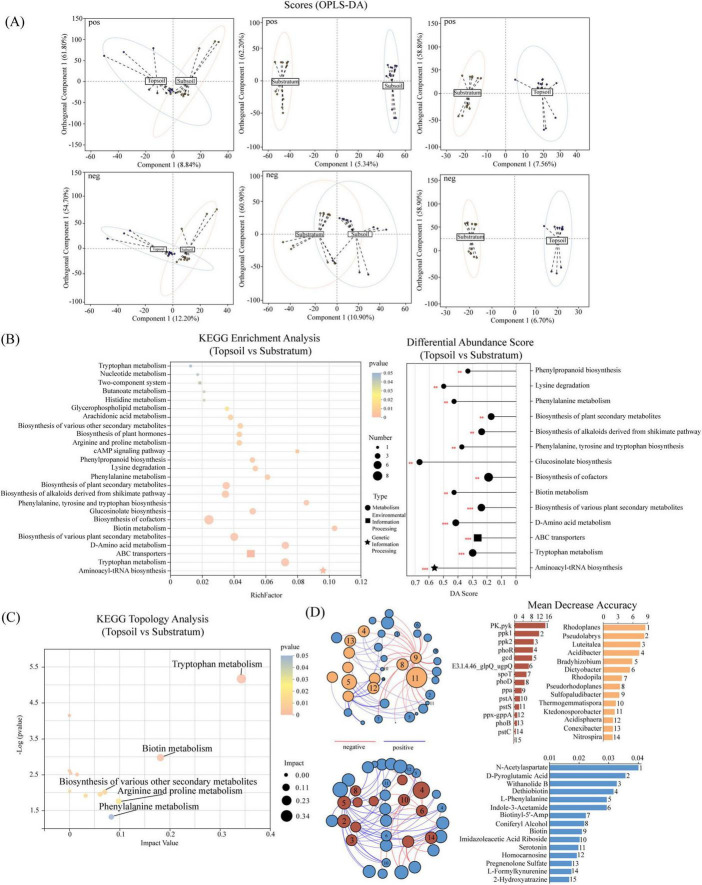
Metabolites reveal soil P cycling pathways and networks. **(A)** OPLS-DA of metabolites across three soil layers. **(B)** KEGG enrichment analysis. Left: KEGG pathway enrichment of differential metabolites. Right: DA scores of KEGG pathways. Bubble color denotes significance, with RichFactor (enrichment ratio) and DA Score (differential abundance) mapped to bubble size. The bubble shape corresponds to KEGG Level 1 categories. The asterisk “*” denotes pathways that are significantly upregulated (positive DA score) or downregulated (negative DA score) in the topsoil relative to the substratum (highly significant: **p < 0.01; extremely significant: ***p < 0.005). **(C)** KEGG topological analysis. The *x*-axis and bubble size represent pathway importance (node influence), while the *y*-axis and color indicate statistical significance. **(D)** Integrated correlation network and random forest analysis of differential metabolites, KOs, and taxa. For random forests, the *x*-axis ranks feature importance. In the network, line color denotes positive (blue) or negative (red) correlations, bubble size reflects relative abundance, and bubble color categorizes factors. Numbers within bubbles correspond to factors from random forest importance rankings.

Investigating the differential distribution and composition of metabolites and their associated metabolic pathways can further elucidate the vertical stratification mechanisms underlying soil P cycling. OPLS-DA modeling revealed distinct differences in both anionic and cationic metabolites exclusively between the topsoil and substratum, suggesting pronounced differences in metabolic pathway activity between the two layers (Figure 3A). Following the removal of irrelevant metabolites, KEGG pathway enrichment analysis was performed on metabolites whose abundance significantly differed (*p* < 0.05) between the topsoil and substratum. The analysis revealed 14 significantly enriched KEGG pathways (*p* < 0.05), predominantly related to metabolism. However, ABC transporters and the cAMP signaling pathway from environmental information processing, along with aminoacyl-tRNA biosynthesis from genetic information processing, were also significantly enriched in the topsoil (Figure 3B). Differential abundance (DA) score plots revealed significant upregulation of these enriched pathways in the topsoil compared with the substratum, confirming the reliance of the topsoil on diverse metabolic strategies to regulate P patterns (Figure 3B). Topological analysis further identified tryptophan metabolism and biotin metabolism as key hubs (node influence values > 0.1, *p* < 0.05) (Figure 3C). They orchestrate multilayered metabolic interactions to synergistically drive P cycling equilibrium in the topsoil. Integrated random forest modeling and cooccurrence network analysis of differentially abundant metabolites, KO terms, and microbial taxa (after weak correlations and low-abundance features were filtered) revealed that among the top 15 influential KOs and microbes, the phoR gene and the taxa *Pseudorhodoplanes*, *Sulfopaludibacter*, *Nitrospira*, and *Ktedonosporobacter* presented prevalent antagonistic relationships with differentially abundant metabolites. In contrast, the majority of the other KOs and microbes presented synergistic associations, suggesting that antagonistic effects may play critical regulatory roles in substratum P patterns (Figure 3D).

### 3.4 Role of abiotic environmental factors in P cycling

**FIGURE 4:**
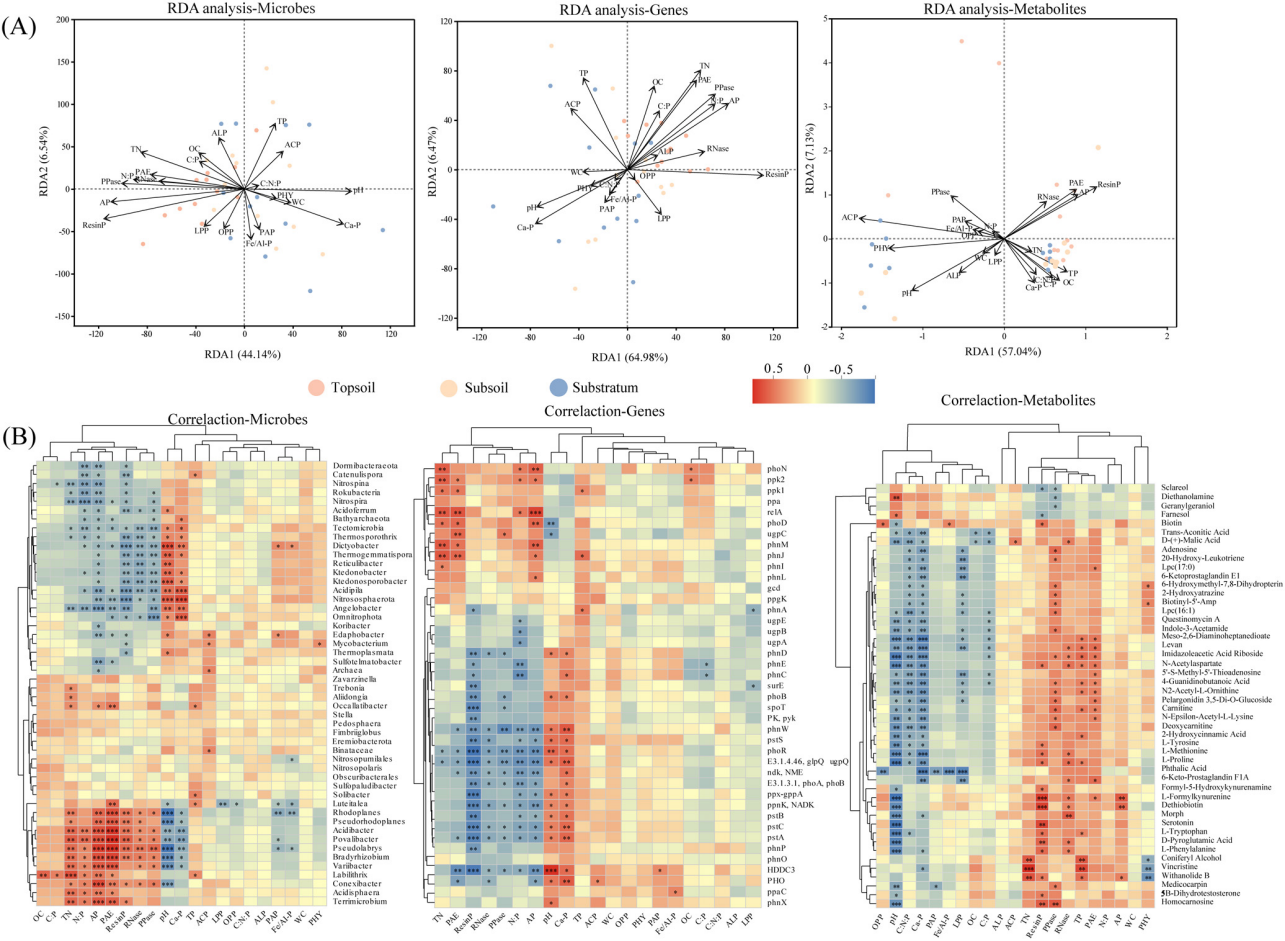
Soil P cycling: Environmental drivers via multi-omics. **(A)** RDA analysis of abiotic environmental factors and multi-omics factors in soil P cycling. **(B)** Correlation heatmap between abiotic environmental factors and multi-omics factors in soil P cycling, with clustering of factors. Correlation coefficients (r) are represented by colored squares, and significance levels are indicated by * (*p < 0.05, **p < 0.01, ***p < 0.001).

P transformation in soils is not directly governed by soil depth but by divergent interactions among environmental factors across soil layers, encompassing both biotic and abiotic components. RDA revealed that low-pH topsoil environments, characterized by elevated C:P, N:P, TN, AP, and PAE ratios, drive microbially mediated P solubilization and mineralization processes (Figure 4A). Correlation analysis (Figure 4B) revealed that differential microbial taxa such as *Acidibacter* and *Bradyrhizobium* were significantly positively correlated with P activation markers (AP, PAE, and Resin-P) and enzymes (PPase and RNase). From an AP-centric perspective, polyphosphate degradation genes (*relA*, *ppk2*) and organic P mineralization genes (*phoD*, *phnL*, *phnM*, *phnJ*, *phoN*) were significantly positively correlated with AP. Additionally, *relA* and *ppk1* were significantly positively associated with PPase and TN. Metabolomics further revealed positive correlations between L-tryptophan derivatives (e.g., formyl-5-hydroxykynurenine) and AP, as well as between biotin-5’-AMP and OPP. The accumulation of 2-hydroxycinnamic acid in the topsoil likely enhances P availability via metal ion chelation, suggesting that multiple pathways have synergistic effects on topsoil P mobilization.

In contrast, the substratum exhibited a distinct P cycling regime marked by higher pH, Ca-P, and PAP (Figure 4A). The pst operon genes (*pstS*, *pstC*, *pstA*, and *pstB*) were negatively correlated with AP but positively linked to Ca-P and pH (Figure 4B). Concurrently, phoR was positively correlated with PAP, which may indicate the activation of high-affinity P uptake and transport mechanisms (e.g., ABC transporters and two-component systems) under P-limited acidic conditions. In the substratum, positive correlations were observed between *HDDC3* and PAP as well as PHY activity and between the microbial genera *Nitrospina* and *Ktedonobacter* and Ca-P. The significant negative correlation between C:N:P stoichiometry and most differentially abundant metabolites underscores the oligotrophic nature of the substratum, constraining metabolic versatility in P activation. Collectively, vertical pH stratification and nutrient stoichiometry may contribute to the functional differentiation of phosphorus cycling across soil horizons, which is mediated by microbe–phosphorus fraction interactions.

### 3.5 Multiomics integration

**FIGURE 5:**
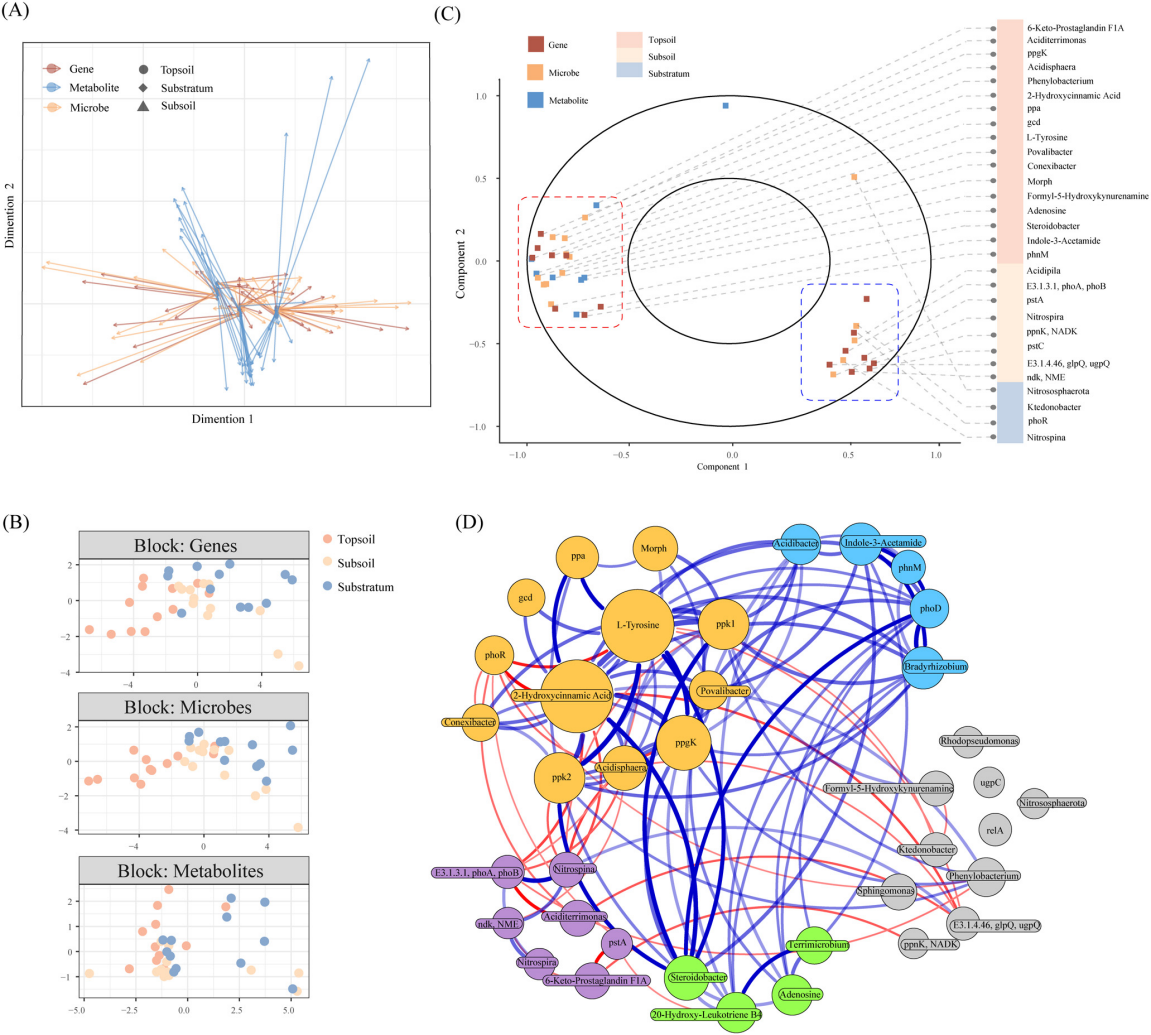
Integrated multi-omics analysis of acidic soil datasets via sPLS-DA and DIABLO. **(A)** Multi-block sPLS-DA arrow plot for three datasets. Arrow origins represent sample centroids across all datasets, while tips indicate dataset-specific deviations. **(B)** DIABLO multi-omics analysis with LefSe-filtered features, visualizing block contributions of samples from three soil layers in latent space. **(C)** DIABLO sample distribution in reduced-dimensional space, highlighting cross-omics associations among layers and biomarker enrichment patterns. **(D)** Cross-omics co-occurrence network. Nodes of the same color belong to one module. Line color denotes correlation direction (blue: positive; red: negative), with thickness and transparency reflecting correlation strength and significance (*p* < 0.05). Weak correlations (*r* < 0.7) were excluded, while key edges were retained.

To reduce the limitations inherent to single-omics analyses, we integrated data from both multiomics and single-omics perspectives. Initial sparse partial least squares discriminant analysis (sPLS-DA) of the interlayer differentially abundant metabolites, KO terms, and microbial taxa clearly separated the three soil layers along Dimension 1 (Figure 5A). The microbial and KO datasets clustered predominantly along Dimension 1, whereas the metabolites diverged along Dimension 2, corroborating previous single-omics inferences and underscoring pronounced stratification and synergistic interactions in acidic soil P cycling. However, this approach neglects crossomics heterogeneity. We thus employed DIABLO to integrate differentially abundant metabolites, KOs, and microbial taxa (Lee et al., 2019). Linear discriminant analysis effect size (LefSe) prefiltered features (LDA > 3, *p* < 0.05) were used to construct a DIABLO matrix. Cross-omics interaction assessment revealed distinct sample clustering in the latent component space, particularly between the topsoil and substratum (Figure 5B). Component 1 effectively discriminated these layers (Figure 5C), with topsoil biomarkers, including adenosine, formyl-5-hydroxykynurenine, indole-3-acetamide, and L-tyrosine, and P-cycling taxa (*Sphingomonas*, *Bradyrhizobium*, *Acidibacter*, and *Terrimicrobium*) alongside their functional genes. The substratum biomarkers included *Ktedonobacter*, *Nitrospina*, *Nitrososphaerota*, and the *phoR* gene. Compared with the substratum and subsoil, the topsoil presented a greater number of multiomics features contributing more to Component 1 (Supplementary Figure 1). Notably, the functional gene *ugpC* and the substratum-dwelling genus *Nitrospina* contributed more than 50% to Component 2 within their respective datasets, whereas presqualene diphosphate in the topsoil demonstrated a negative contribution exceeding 80%. These findings suggest that diverse microbial taxa in the topsoil rely on multifaceted metabolic pathways to maintain P allocation, extending beyond the roles of acidophiles and nitrifiers.

Functional co-occurrence networks integrating DIABLO-derived biomarkers revealed a modular architecture (modularity = 0.326, Figure 5D) with five distinct modules (Mushtaq and Fauconnier, 2024). The gray and purple modules showed broad antagonism with the largest module, whereas the green and blue modules exhibited synergy. The module highlighted positive correlations between topsoil-enriched taxa (Conexibacter, Povalibacter, and Acidisphaera), polyphosphate metabolism genes, inorganic P solubilization genes, and metabolites (2-hydroxycinnamic acid and L-tyrosine), implicating these compounds as potential hubs in topsoil P cycling. Intriguingly, phoR (substratum-enriched) antagonized the largest module factors. The gray and purple modules emphasized P-cycling taxa of substrata (*Ktedonobacter*, *Sphingomonas*, and *Aciditerrimonas*) synergizing with nitrogen-cycling (*Nitrospira*, *Nitrospina*, and *Nitrososphaerota*) and carbon-cycling (*Phenylobacterium*) taxa. This may suggest that substratum microorganisms maximize resource utilization through nitrification and acid production pathways under nutrient-limited conditions. Notably, the DIABLO network reiterates the importance of formyl-5-hydroxykynurenine (a tryptophan degradation intermediate) and *relA* (activator of the tryptophan operon) in P cycling, underscoring their critical roles. Targeted experiments are warranted to dissect how tryptophan metabolites modulate P activity, P speciation, and P-solubilizing microbial abundance in acidic soils.

**FIGURE 6:**
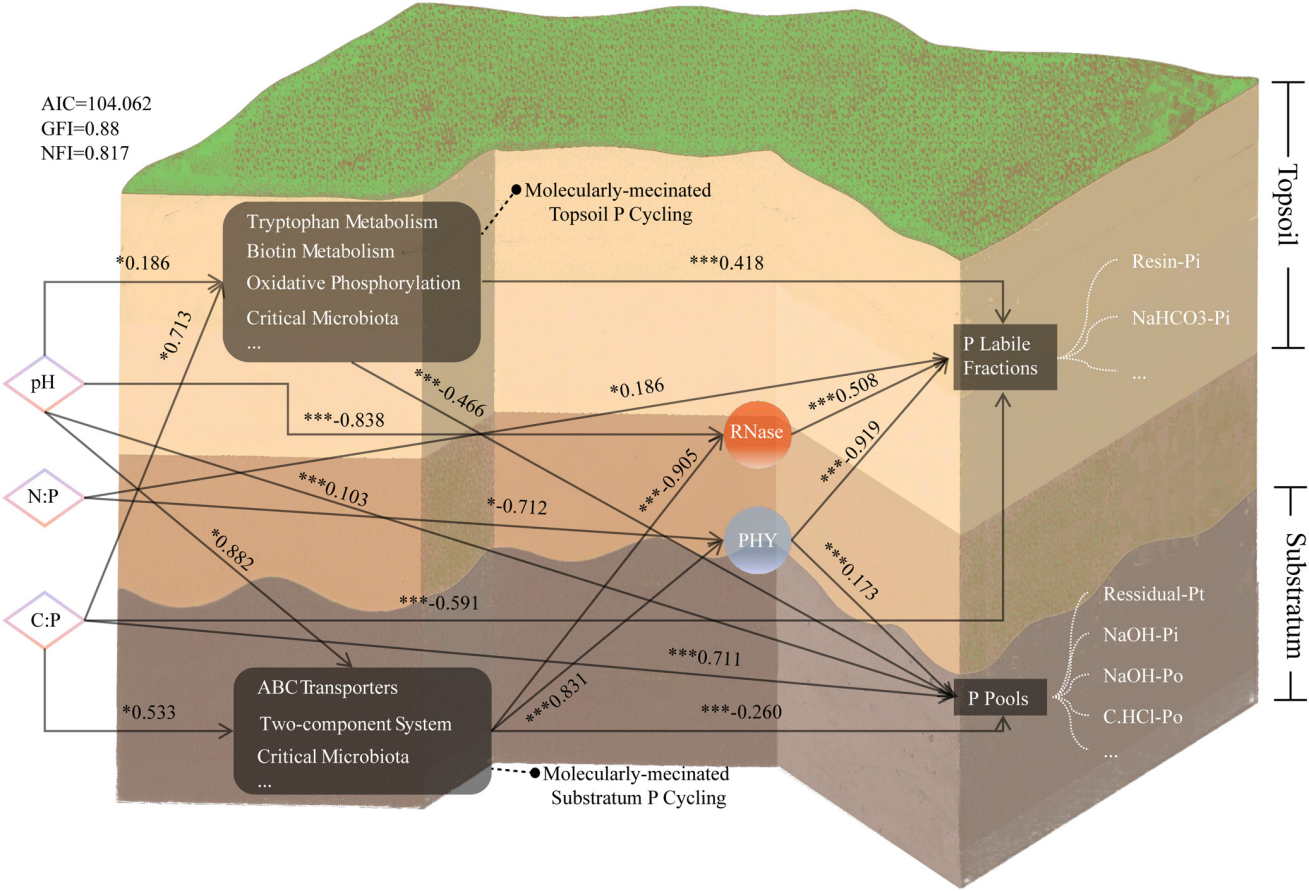
A cross-layer SEM based on DIABLO analysis reveals causal interactions among canonical abiotic environmental factors, two P indices, and biomarkers. Asterisks denote statistical significance: *p < 0.05 (significant), ***p < 0.005 (extremely significant).

To quantify the contributions of abiotic environmental factors, microbial metabolism, and their interactions with P cycling, we conducted structural equation modeling (SEM) (Figure 6) on feature datasets and abiotic factors. P metrics were categorized into labile P fractions and P pools, with dimensionality reduction via principal component analysis (PCA). Dimensionality reduction via PCA was separately performed on biomarkers of key pathways in the topsoil and substratum. DIABLO-integrated results revealed that elevated pH and the C:P ratio (but not the N:P ratio) positively influenced P-activating microbes and associated metabolism, with pH exerting the strongest effect on substratum molecular mechanisms (path coefficient = 0.882). pH also directly suppressed RNase activity (–0.838) and altered P speciation. The C:P ratio had a secondary influence on the topsoil (0.713). The topsoil microbiota affected P transformation through diverse pathways, resulting in stronger effects on labile P (0.418) and P pools (–0.466). In contrast, substratum microbes rely on limited strategies, such as PHY (0.831), to decompose recalcitrant P with a relatively low impact on P pools. Our findings demonstrate associations between distinct metabolic mechanisms and the vertical stratification of phosphorus cycling across the soil.

## 4 Discussion

The stratified cycling of soil P represents a contradiction; however, plant-available P generally decreases with increasing soil depth (Ding et al., 2014; Shao et al., 2019), and the mechanisms affecting the vertical differentiation of P fractions remain poorly understood, particularly in acidic soils with limited labile P. This study elucidates the vertical stratification of P cycling in acidic soils. The topsoil enhances P availability through high microbial diversity and synergistic metabolic pathways, whereas the substratum maintains P homeostasis via the Pst operon and two-component regulatory systems, albeit with low functional redundancy and simplified transformation pathways. These microbial-metabolic stratifications are partially mediated by vertical gradients in pH and nutrient stoichiometry. Our findings provide actionable insights for optimizing topsoil fertilization and substratum amendments in ferralsols.

### 4.1 Fertilization and dry-season irrigation modulate P cycling

Multiple studies have demonstrated that irrigation reshapes the soil microbial composition (Huo et al., 2024), enhances bacterial–fungal network connectivity (Upton et al., 2020), and modifies P fractions (Upton et al., 2020), generally promoting P cycling. Similarly, Lüneberg et al. (2018), nutrient amendments alter P speciation. Owing to the low mobility of P (Lüneberg et al., 2018), P fertilization increases labile P accumulation in the topsoil (Abdi et al., 2019). N addition indirectly affects P by lowering pH and altering microbial biomass and litter inputs rather than directly modifying P fractions (Liebig et al., 2002; Messiga et al., 2014; Li et al., 2021). However, our findings strongly contrast these paradigms. In our study, neither irrigation nor fertilization nor their interactions had significant effects on P activation, P-cycling microbial communities (Table 3), or functional gene expression (Table 4), at least during the dry season. Notably, while dry season irrigation was applied annually, fertilization ceased after 4 years. This discrepancy may have arisen because dry season irrigation failed to increase soil moisture thresholds across the plantation, necessitating further experiments to quantify moisture gradient effects on labile P migration. Fertilization transiently increases surface nutrient levels, but its impact on tree uptake and long-term soil nutrient cycling is context dependent (Binkley and Högberg, 2016; Zhang et al., 2021). In our system, natural P migration over 3 years offset labile P accumulation from 4 years of fertilization (Huo et al., 2024), a predictable outcome given that persistent P leaching is attributable to prolonged rainfall and groundwater fluctuations (Wang et al., 2023; Chen et al., 2025), particularly for plant-available P. Fertilization’s limited capacity to replenish labile P underscores the need to quantify the lag effects of P inputs on acidic soil P pools (Liang et al., 2016), informing the precision management of plantation P fertilization. A limitation of this study is that sampling and analysis were conducted only during the dry season. Previous research indicates that soil P may vary with treatment during the rainy season (Turner and Haygarth, 2001) and that its stratification could be more pronounced due to strong leaching (Chen et al., 2022). Consequently, based on long-term monitoring data, we will develop a climate dynamic model for P fractions across soil layers (Yang et al., 2019) to assess the sustainability of the soil P pool and to further inform plantation cultivation practices.

### 4.2 Multiomics synergistic regulation of P across soil layers

As hypothesized, we identified distinct multiomics networks affecting P cycling in the topsoil and substratum. Tryptophan, a critical component of microbial proteins and a precursor for growth regulators and secondary metabolites (Barik, 2020; Bĕlonožníková et al., 2022), plays a central role in N metabolism while fueling carbon metabolism via degradation-derived substrates (Wei et al., 2025). Our multifaceted analyses consistently revealed connections between tryptophan metabolism and topsoil phosphorus transformation (Figures 2E, 3C, 6). Soil microbes likely leverage this pathway to produce metabolites that solubilize recalcitrant P (Rajkumar et al., 2005; Bispo et al., 2023), stimulating the proliferation of phosphate-solubilizing bacteria and P activation (Figure 4B), which is supported by significant correlations between tryptophan metabolism-related KOs/metabolites and bioavailable P fractions (Supplementary Table 2; Cheng et al., 2024). These findings warrant experimental validation (e.g., exogenous L-tryptophan supplementation) to validate its role in topsoil P release. Biotin, a coenzyme for carboxylases involved in CO*2* fixation and carbon metabolism (Lietzan et al., 2014), was also enriched in the topsoil (Figure 3C). While its importance in aquatic systems has been established (Panzeca et al., 2006; Xu et al., 2022), its role in microbial P metabolism remains underexplored. We propose that diverse P-cycling pathways in topsoil may depend on energy provisioning from biotin metabolism and tryptophan metabolism. For key tryptophan metabolites, we will subsequently employ δ^18^O-P-labeled phosphate and ^13^C-labeled tryptophan to trace *in situ* the translocation of active P components from topsoil to subsoil (Tamburini et al., 2012; Amelung et al., 2015), clarifying the C–P linkage in tryptophan metabolism. On the other hand, we consider it necessary to separately sample rhizosphere and bulk soils from different soil layers. By comparing multi-omics profiles, we aim to determine whether plantation roots recruit specific subsoil P-solubilizing microorganisms (Walker et al., 2003) or alter metabolic activities of indigenous soil microbes by root exudates (Spohn et al., 2013.), thereby acquiring P from the P pool.

The oligotrophic substratum, with extremely low P availability (Figure 1C; Weihrauch and Opp, 2017), forces bacteria to evolve adaptive strategies (White and Metcalf, 2007). The *PhoB*-*PhoR* two-component system regulates phosphate (Pi) sensing and uptake: PhoB activates ABC transporters via DNA binding, enabling ATP-driven Pi transport (Chakraborty et al., 2011; Bisson et al., 2017). Despite systemic P scarcity, topsoil microbes downregulated *phoR*, *phoB*, and *phoA* (Figure 2D), likely due to elevated labile P from fertilization and dry-season irrigation and litter decomposition (Figure 1C). In ferralsols, labile P is readily immobilized by iron and aluminum oxides, resulting in significantly reduced bioavailability (Lammel et al., 2018), driving topsoil microbes toward alternative P acquisition (Wu et al., 2023; Zhao X. et al., 2025). Notably, ABC transporters were enriched in the topsoil alongside upregulated *ugpC*, whereas low-P adaptation genes (pstS, pstB) were downregulated (Figures 2D, 3D). These findings indicate that the substratum microbiota has adaptively evolved under extreme Pi-limiting conditions, albeit at the expense of P metabolic diversity, an inference congruent with our prior studies.

### 4.3 Cross-stratum interactions in P cycling

Our prior studies revealed vertical stratification in acidic soil P cycling, with topsoil and substratum P coupled through physical migration, microbial metabolic interactions, and abiotic environmental factors, resulting in cross-stratum synergy (Figures 5D, 6) and functional division (Pan et al., 2023). First, cross-stratum microorganisms establish a “functional relay” for P transformation (Liu Z. et al., 2024). Topsoil taxa such as *Acidisphaera* and *Aciditerrimonas* may release bound P by secreting phenolic acids to chelate the substratum Fe^3+^ (Honeker et al., 2019) while suppressing phoR histidine kinase autophosphorylation in the substratum (Monds et al., 2006). Conversely, substratum *Nitrospira* enhances Fe/Al-P reactivation in the topsoil via nitrification-driven pH reduction, an interaction reflected in the antagonistic relationship between the largest module and purple module in the DIABLO network (Zhao X. et al., 2025). Despite minimal ALP activity (Figure 1B), its positive correlation with AP and PAE (Figure 4B) suggests microbial reliance on ALP-mediated mineralization under neutral conditions. However, acidic topsoil forced the microbiota to prioritize ACP and phosphoester degradation for organic P mineralization. Labile P leaches downward during rains, becoming immobilized as Fe/Al-P in the substratum (Figure 1C) or lost via runoff (Schoumans and Groenendijk, 2000). Substratum *Nitrospira* may partially solubilize Ca-P (Zhang et al., 2019), with released Pi migrating upward via deep-root uptake (Lehmann, 2003), mycorrhizal networks, or capillary action (Jorenush and Sepaskhah, 2003). Our study demonstrated that topsoil tryptophan metabolism may stimulate the substratum xerotolerant bacterium *Ktedonobacter* to utilize alternative P sources (Cheng et al., 2024), thereby promoting the degradation of phosphoester-derived Po (Figure 5D).

Although microbial communities differ markedly between layers (Figure 2A), some functional genes exhibit vertical complementarity via polyphosphate (polyP) metabolism. The gcd-encoded glucose dehydrogenase (GCD) releases protons during oxidation, lowering the pH to solubilize mineral phosphates, a process reflected in gcd’s role as a predictor of the AP content (Li et al., 2019). However, the lack of correlation between gcd and AP in acidic topsoil (Figure 4B) may indicate GCD inefficiency at low pH, driving microbes toward alternative solubilization strategies. The enzyme encoded by relA synthesizes (p)ppGpp, triggering a bacterial stringent response and activating phosphatase synthesis under P starvation. Additionally, (p)ppGpp inhibits polyphosphatase (PPX) activity, reducing polyP degradation and thereby assisting microbial intracellular P reserve maintenance in fluctuating P environments (Rao et al., 1998; Hamm and Gray, 2025). Polyphosphate kinase 2 (PPK2), encoded by ppk2, catalyzes polyP synthesis and regeneration as an intracellular P reserve (Rao et al., 2009). The significant expression of ppk2 and relA in topsoil further suggests low-P and low-pH stress in this layer, with both genes synergistically synthesizing polyP to reduce Pi fixation with Fe^3+^ and Al^3+^ (Figure 1C), maintaining P homeostasis. Substratum microbes, which are exposed to relatively high pH and extremely limited labile P, must sustain P homeostasis and basal energy metabolism while finding alternative P sources. Under substratum oligotrophic conditions, nucleoside–diphosphate kinase facilitates the interconversion of nucleoside diphosphate (NDP) and triphosphate (NTP), stabilizing intracellular energy (ATP) and the nucleotide pool to balance P acquisition and energy metabolism (Nitschmann and Peschek, 1986). Elevated expression of NAD^+^ kinase genes in the substratum (Figure 2D) likely enhances NADP^+^ synthesis, protecting P-metabolizing enzymes by improving microbial resistance to metal ion oxidation. Overall, upregulated substratum gene expression may indicate guanosine-3’, 5’-bis(diphosphate) (ppGpp) accumulation (Figure 2D; Chakraborty et al., 2011). Thus, ppGpp accumulation promotes polyP hydrolysis to release Pi, sustaining P metabolism under extreme P deficiency. Elevated expression of NAD^+^ kinase genes in the substratum (Figure 2D) likely enhances NADP^+^ synthesis, protecting P-metabolizing enzymes by improving microbial resistance to metal ion oxidation (Oka et al., 2023). A seemingly contradictory finding is the significant upregulation of HDDC3 in the substratum versus the topsoil (Figure 2D; Spira and Yagil, 1998). HDDC3 hydrolytically degrades ppGpp (Büke et al., 2022). Given the dual scarcity of labile P and carbon sources in the substratum, HDDC3 upregulation may represent a bacterial strategy to balance Pi release with energy expenditure, preventing the suppression of non-P metabolic pathways during prolonged P starvation (Gui et al., 2022).

## 5 Conclusion

This study validated a “surface activation-substrate retention” vertical stratification pattern of soil P cycling during the dry season under subtropical plantation management practices via integrated multiomics analysis, revealing potential linkages among abiotic factors, multiomics metabolic networks, and phosphorus stratification. The key findings are summarized as follows:

(1) Vertical stratification patterns of P cycling: Topsoil presented significantly greater P availability than substratum did, characterized by elevated ACP activity and enrichment of diverse P-solubilizing functions. In contrast, the substratum maintained P homeostasis, with dominant insoluble P forms.

(2) Key regulators and pathways: pH and the C:P ratio are critical factors in the vertical differentiation of soil phosphorus cycling. Under low pH conditions, topsoil microorganisms contribute to P release through diverse metabolic pathways (potentially including tryptophan metabolism). Substratum oligotrophic conditions constrain enzymatic activities (e.g., pyrophosphatase), whereas microbial communities prioritize the optimization of P uptake efficiency.

(3) Multiomics regulatory network: We identified potential cross-strata microbial functional synergies in soil P cycling within *Eucalyptus* plantations. Topsoil microorganisms may mobilize substratum P through phenolic acid secretion, whereas substratum microbes balance P acquisition with energy metabolism. DIABLO analysis revealed polyP metabolism as the functional nexus.

Our study demonstrated that the effect of dry-season irrigation and the residual effect of fertilization had a limited influence on labile P during the drought period. Future research on ferralsols should prioritize exogenous regulation of tryptophan metabolic pathways and elucidate roote drought period. Future research on ferralsols to increase long-term P utilization efficiency and soil P sustainability.

## Data Availability

The datasets presented in this study can be found in online repositories. The names of the repository/repositories and accession number(s) can be found at: https://www.ncbi.nlm.nih.gov/bioproject/?term=PRJNA1363667; https://www.ebi.ac.uk/pride/archive/projects/PXD070738.

## Footnotes

1
http://www.bioinformatics.org/cd-hit/

2
https://anaconda.org/channels/bioconda/packages/soapaligner/files

3
https://www.kegg.jp/kegg/mapper.html
