# Supplementary material for: Vertical stratification of P pools in subtropical plantation soils under fertilization and dry–season irrigation: multiomics regulatory strategies
Source: Front Microbiol. 2026 Jan 5;16:1714023. doi: 10.3389/fmicb.2025.1714023 (PMC12812563; doi:10.3389/fmicb.2025.1714023)
Supplement: Supplementary file 1 [file Data_Sheet_1.docx]

**Table S1** Phosphorus indices related to soil phosphorus fractions

| Index of phosphorus cycle | Calculation method | Abb. | Ecological significance |
| --- | --- | --- | --- |
| Resin-Pi | - | - | Highly labile Pi |
| D-HCl-P | - | Ca-P | Calcium phosphate |
| available phosphorus | Resin-Pi+NaHCO3-Pi | AP | Short-term soil phosphorus supply |
| iron and aluminum-bound phosphorus | NaOH-Pi+NaOH-Po | Fe/Al-P | Important source of availability phosphorus in acidic soil |
| Labile phosphorus pool | Resin-Pi+NaHCO3-Pi+NaHCO3-Po | LPP | Short-term soil phosphorus flux |
| Phosphorus activation efficiency | (Resin-Pi+NaHCO3-Pi)/(Resin-Pi+NaHCO3-Pi+NaHCO3-Po) | PAE | The ability of insoluble phosphorus conversion to bioavailable |
| Potentially available phosphorus | Resin-Pi+NaHCO3-Pi+NaHCO3-Po+NaOH-Pi | PAP | Reserves of long-term phosphorus supply |
| Organic phosphorus mineralization potential | NaHCO3-Po+NaOH-Po+C.HCl-Po | OPP | The ability of organic phosphorus into inorganic phosphorus |
| Total phosphorus | Sum of all phosphorus components | TP | Represents phosphorus stock |
| Carbon to Phosphorus Ratio | OC/total phosphorus | C:P | Regulation of microbial mineralization-stabilization balance​ |
| Nitrogen to Phosphorus ratio | TN/total phosphorus | N:P | Regulation microbial-mediated phosphorus transformation |
| Soil Organic Carbon to Nitrogen to Phosphorus Ratio | OC/TN/total phosphorus | C:N:P | Assessing soil nutrient limitations and microbial activities |

Note: Pi, inorganic phosphorus; Po, organic phosphorus; C.HCl-Pi/Po, concentrated HCl extracted inorganic/organic phosphorus.

**Table S2** Correlation analysis between tryptophan metabolism-related factors and phosphorus cycle indicators

| Index | ALDH | ACAT, atoB | GCDH, gcdH | DLD, lpd, pdhD | E3.5.1.4, amiE | DDC, TDC |
| --- | --- | --- | --- | --- | --- | --- |
| Resin-Pi | -0.408* | -0.394* | -0.474** | -0.347* | -0.525** | -0.507** |
| AP | -0.156 | -0.094 | -0.153 | 0.071 | -0.297 | -0.366* |
| PAE | -0.167 | -0.200 | -0.267 | -0.198 | -0.367* | -0.370* |
| RNase | -0.134 | -0.104 | -0.375* | -0.186 | -0.301 | -0.444** |
| TN | -0.171 | -0.181 | -0.226 | -0.128 | -0.328 | -0.433** |
| N:P | -0.242 | -0.235 | -0.292 | -0.146 | -0.432** | -0.518** |
| Fe/Al-P | 0.395* | 0.204 | 0.082 | 0.196 | 0.178 | 0.008 |
| C:N:P | 0.364* | 0.367* | 0.159 | 0.294 | 0.303 | 0.184 |
| ALP | 0.294 | 0.095 | 0.351* | 0.164 | 0.331* | 0.351* |

Note: Pi, inorganic phosphorus; Po, organic phosphorus; C.HCl-Pi/Po, concentrated HCl extracted inorganic/organic phosphorus.

| 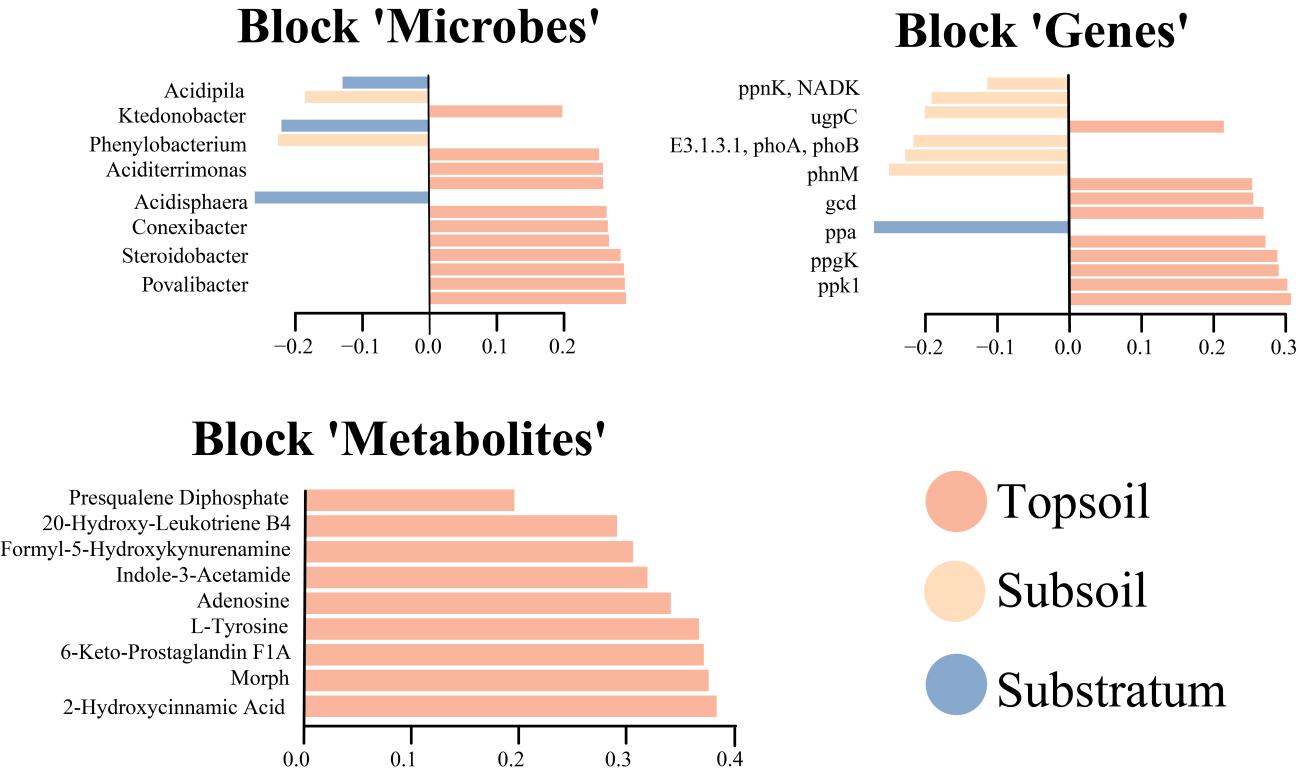 |
| --- |
| **Figure S1**  Contributions of Multi-Omics Factors from Different Soil Layers to Principal Component 1 |
